# Nanoliposome loaded with 5-fluorouracil and Dorema aucheri extract reduces tumor growth in a mouse model of colorectal cancer

**DOI:** 10.1038/s41598-025-18676-6

**Published:** 2025-10-07

**Authors:** Fatemeh Keshavarz, Firouzeh Nasimi, Hassan Bardania, Mohsen Soltanshahi, Kamran Goudarzi, Erfan Azadifar, Niloufar Mohamadi, Parisa Ramiyan, Ghasem Ghalamfarsa

**Affiliations:** 1 Student Research Committee, Department of Immunology, Shahid beheshti University of medical scieneces, Tehran, Iran; 2 Department of Medical Biotechnology, Shahid Sadoughi University of Medical Sciences, Yazd, Iran; 3 Department of laboratory science, faculty of paramedicine, Yasuj University of Medical Sciences, Yasuj, Iran; 4 Cellular and Molecular Research Center, Yasuj University of Medical Sciences, Yasuj, Iran

**Keywords:** Cancer, CRC, Nanoliposomes, Dorema aucheri, 5-fluorouracil, Biochemistry, Biotechnology, Cancer

## Abstract

The rising incidence of colorectal cancer (CRC) and the limited success of conventional therapies have spurred efforts to identify promising new drugs with fewer side effects. Dorema aucheri (DA) has shown potential as An Anti-cancer agent. Additionally, 5-fluorouracil (5-FU), a chemotherapy drug widely used for CRC. This study aims to investigate the effect of Liposome-encapsulated 5-FU and DA on tumor growth in a mouse model. The lipid thin-film hydration method was used to create the liposomes, and high-performance liquid chromatography (HPLC), dynamic light scattering (DLS), and transmission electron microscopy (TEM) were used to evaluate their characteristics. Using an MTT experiment, the impact on cell growth was assessed. BALB/c mice were implanted with CT26 mouse tumor cells. Tumor growth was monitored by measuring tumor size. Real-time PCR was used to look at the levels of expression of VEGF, VEGF-R2, VE-Cadherin, VEGF-C, and β-actin genes in tumor tissue. The nanoparticles that were developed exhibited a spherical shape, uniform size, And An average diameter of 146 ± 4.6 nanometers. HPLC Analysis demonstrated that approximately 80% of the compound was successfully encapsulated within the nanoliposomes. The MTT assay results showed that the nanoliposome-encapsulated 5FU + DA (NLP + 5FU + DA) formulation resulted in a dose-dependent reduction in cell proliferation. The group treated with NLP + 5FU + DA exhibited the slowest tumor volume growth and the highest weight gain. Furthermore, the expression levels of VEGF, VEGF-R2, VE-Cadherin, and VEGF-C genes in the tumor microenvironment Comparing the NLP + 5FU + DA group to the control group revealed a substantial decrease. The combination of nanoliposomes loaded with 5-FU and DA can reduce the growth rate of colorectal tumors in mice. It can also reduce the expression of VEGF, VEGF-R2, VE-Cadherin, and VEGF-C genes.

## Introduction

The most common type of gastrointestinal cancer worldwide is colorectal cancer (CRC), which starts in the colon or rectum^1^. In the United States, approximately 150,000 new cases of CRC are diagnosed each year, with around 35% of those individuals succumbing to the disease^2,3^. While the precise cause of CRC remains unclear, it is widely believed that multiple factors contribute to its development, and existing evidence strongly associates certain risk factors with the disease^4^. For instance, genetic predispositions, lifestyle choices (such as diet, smoking, and alcohol use), and a history of conditions Like inflammatory bowel disease, obesity, And type 2 diabetes are significantly linked to an increased risk of developing CRC^5–7^. CRC can be treated using a variety of approaches, including surgery, chemotherapy, radiation, And immunotherapy. However, patients diagnosed with advanced-stage CRC have a very low survival rate, around 5%, even following surgical intervention^8,9^.

5-Fluorouracil (5-FU) is the standard chemotherapeutic drug for CRC treatment and belongs to the class of synthetic fluorinated pyrimidine analogs^10^. Despite recent breakthroughs in therapeutic techniques, the 5-year survival rate for CRC patients is quite low^11^. In cases of metastatic CRC, over 90% of patients develop inherent or acquired resistance to treatment. Some important mechanisms contributing to 5-FU resistance involve epithelial-mesenchymal transition, epigenetic alterations, and gene expression changes^12^. Although less toxic, 5-FU toxicity and severe adverse effects have been reported in patients with CRC, which include fever, fatigue, mucositis, stomatitis, nausea, vomiting, and diarrhea. Other possible toxicities are leukopenia, neutropenia, thrombocytopenia, anemia, neuropathy, and cutaneous toxicity. Neurological adverse effects, like cerebellar ataxia and cognitive dysfunctions, are uncommon but not absent in all patients^13^.

The Bilhar plant, scientifically known as Dorema aucheri (DA), belongs to the Chetrian family and is found in certain regions of Iran, where it is used for its nutritional value. Research indicates that this plant may possess anti-cancer properties. Specifically, studies have demonstrated that the high concentration of Caryophyllene in the oil of DA exhibits cytotoxic effects on colorectal cancer cells, such as SW48 and SW1116^14^. Other investigations have also highlighted the antimicrobial and cytotoxic potential of the plant’s extract^15,16^. Available evidence suggests that Bilhar extract is rich in antioxidant compounds, including flavonoids, anthocyanins, phenolic acids, and a variety of terpenoids^17^. These bioactive compounds, particularly flavonoids, are believed to contribute to the extract’s ability to neutralize free radicals, thus enhancing its antioxidant properties^18^. This is the first study on the combination of a plant And 5FU, but previous studies have already shown that herbal medicines can sensitize resistant treatment cells.The combination of herbal medicines And 5FU can sensitize resistant treatment cells, with phytochemicals like curcumin, resveratrol, EGCG, genistein, geraniol, and thymoquinone enhancing cytotoxicity and inhibiting resistance. Corosolic acid and oxymatrine reverse multidrug resistance, enhancing the therapeutic effect^19–23^.

Chemotherapy and radiotherapy, combined with medication resistance and toxicity, have a significant effect on the quality of life and prognosis of colorectal cancer patients. In this regard, liposomes are a promising strategy for targeted cancer therapy that can be used to enhance safety and efficacy in medication treatments^24–26^. Liposomes are vesicles composed of lipids that are capable of encapsulating hydrophilic and hydrophobic molecules. They exhibit low toxicity, are biodegradable, and are biologically compatible. To address these issues, researchers have concentrated on developing nanoparticle-based medication delivery methods^27–29^.

Developing innovative treatment techniques is essential given the high incidence of colorectal cancer, its accompanying consequences and death, and our growing understanding of the disease’s underlying causes. Since many chemotherapy medicines are cytotoxic, nanoliposomes present a viable method for more precisely and effectively delivering these medications to the tumor’s location^30^. Given that DA exhibits antioxidant activity, which may aid in reducing oxidative stress and potentially alleviating some chemotherapy side effects, and that it has demonstrated anticancer effects in our previous studies^31,32^, it could potentially enhance the efficacy of 5-FU through synergistic effects. This study, for the first time, investigated the Anti-tumor effects of two compounds, DA And 5-FU, loaded in liposome nanoparticles on a mouse model of colorectal cancer.

## Supplies and techniques

### Supplies

Cholesterol (Cat. No. C8667-25G), MTT assay reagents (11 465 007 001), phospholipid DPPC (850355P) were obtained from Sigma-Aldrich (Germany). Pen/Strep (15070063), RPMI 1640 medium (31800089), FBS (26140079) and PBS (18912014) were obtained from Gibco (USA). Chloroform (1024441000) And 5-fluorouracil (818505) were obtained from Merck (Germany). Xylazine (36408/3007) and ketamine (36408/3000) were obtained from Alfasan (Sofia, Bulgaria), Biochemical Factor Measurement Kit was obtained from Bionik (Iran).

### Apparatus

The zeta potential (ZP) of the liposomes was measured using a Zetasizer Nano ZS device from Malvern Instruments. A Bio-Tek Instruments ELX800 UV universal microplate reader for ELISA tests, a Bio-Rad thermocycler for PCR, a KNAUER (Germany) high-performance liquid chromatography (HPLC) system, and a Philips CM30 transmission electron microscopy (TEM) for imaging were among the additional tools utilized. Nanodrop ND One model from THERMO FISHER (USA). Automated biochemistry analyzer (Roche COBAS, Mira Plus, USA) for biochemical tests.

### Extract preparation

Early in the spring, aboveground portions of DA (Bilhar) were gathered from rural regions close to Yasuj in the Iranian provinces of Kohgiluyeh and Boyer-Ahmad. The hydroalcoholic extract of Bilhar was made using these specimens. The Herbarium of the Medicinal Plants Research Center at Yasuj University of Medical Sciences verified the identity of the plants (voucher number. 0496). The gathered samples were crushed into a fine powder after being allowed to air dry in a shady area. Following that, 1,200 milliliters of 70% ethanol were used to macerate 300 g of the powdered plant material for 72 h at room temperature. After filtering and centrifuging the mixture, the supernatant was removed by evaporating it. At −4 degrees Celsius, the finished extract was kept in storage. To get the correct concentrations, the necessary amount of extract was dissolved in 80% methanol^33^.

### Nanoliposomes synthesis

The Lipid thin-film hydration approach outlined in a prior study was used to create nanoliposomes loaded with 5-FU and DA (NLP + 5FU + DA)^34^. First, precise molar ratios of 3:7 were used to dissolve lyophilized phospholipids, such as cholesterol And distearoylphosphatidylcholine, in chloroform. Chloroform was evaporated using a rotary evaporator set to 50 °C And 150 rpm while under vacuum to create a Lipid thin layer. Over the course of two hours, the Lipid film was progressively moistened with a PBS solution that contained 2 mg of 5-FU and DA (2 mg in 500 µl of methanol). Any free, unencapsulated medications were extracted by centrifugation at twenty-nine thousand g for 30 min after the resultant nanoliposomes were sonicated to create tiny unilamellar vesicles.

### Characterizations

Transmission electron microscopy (TEM; Philips CM30, Netherlands) was used to visualize the morphological and structural features of the resultant nanoliposomes (NLPs). For TEM imaging, a tiny amount of the homogenized and diluted NLP formulation was applied on carbon-coated copper grids (400 mesh; Agar Scientific, UK). In order to investigate the uniform sphericity of nanoliposomes, a few randomly selected fields or sections of different prepared samples were imaged in order to avoid selection bias and ensure representativeness. Individual nanoliposomes (at least 100) for each field were analyzed for the parameters of diameter, aspect ratio, and sphericity index. Mean and standard deviation of sphericity indices and aspect ratios for each field were calculated. These parameters were compared between fields using ANOVA.

Using dynamic light scattering (DLS) analysis (Malvern Zetasizer Nano ZS Instruments) with a 633 nm argon laser And a 90° scattering Angle, the average particle size And zeta potential of the diluted NLPs were determined. Two milliliters of NLPs dissolved in distilled water were used for the measurements, which were carried out at 25 °C. The NLPs’ size And drug release were tracked for four weeks at 4 °C in order to assess their stability. DLS was used to determine particle size.

### Encapsulation efficacy

The encapsulation efficiency (EE%) of NLP-5FU-DA was determined by high-performance liquid chromatography (HPLC) using a KNAUER system (Germany). Centrifugation was performed for the drug-loaded Liposome formulations to phase out the drug entrapped pellet, which was again resuspended in methanol And sonicated for 10 min to release the drug entrapped 5-FU and DA. Chromatographic separation was carried out on a C18 analytical column (4.6 mm × 150 mm, particle size 5 μm) at 25 °C. The injection volume was 10 µL, And mobile phase flow was done at a flow rate of 1.0 mL/min. In the determination of 5-FU, the mobile phase was made up of 95% acidified water (pH 3.5 with phosphoric acid) And 5% methanol And detected at 265 nm. Detection of DA, however, was with a mobile phase made up of 52% 0.4% acetic acid And 48% acetonitrile And detected at 430 nm. Drug entrapment efficiency (DEE) was calculated by using following equation:

$$DEE=C_{0}-C_{1} /C_{0}\times100.$$

where C0 is initial concentration of drug and C1 represent nonentrapped drug in supernatant.

### Release assay

The release of 5-FU And DA from nanoliposomes under laboratory conditions was evaluated using the dialysis bag diffusion technique. A volume of 1 milliliter of the nanoliposome suspension containing specific amounts of 5-FU and DA was placed in a pre-soaked dialysis bag (MWCO 12–14 kilodalton). The sealed bag was submerged in 50 milliliters of PBS (pH 7.4) for 5-FU or PBS: ethanol (1:1) for DA And gently shacked in a water bath at 37 degrees Celsius under stirring (100 rpm). Every 0.5, 1, 2, 4, 6, 8, 12, 24, 48, And 72 h, 1 milliliter of the released culture medium was removed And the same amount of fresh buffer was added immediately to maintain sink conditions. The samples taken were Analyzed for absorbance using a UV-Vis spectrophotometer at 266 nanometers for 5FU And 425 nanometers for DA. Finally, percentage drug release was computed in total and plotted vs. time to determine the release kinetics. All experiments were performed in triplicate, and data were expressed as mean ± standard deviation.

### Hemolysis assay

biocompatibility was evaluated with a hemolysis test. Fresh human venous blood was prepared in a heparinized tube. 2 milliliters of the whole blood sample were centrifuged at 10,000 g for 5 min to separate the red blood cells (RBCs). The red blood cells were washed three times with PBS after separation and finally resuspended in PBS. Different concentrations of nanoliposomes (2.5, 12.5, 62.5, 125, 250, or 500 µg/ml) were prepared in PBS. Then, 0.2 milliliters of the diluted red blood cell suspension were exposed to 0.8 milliliters of the nanoparticle suspension to achieve final nanoparticle concentrations of 2, 10, 50, 100, 200, or 400 µg/ml (experimental group), distilled water (positive control), and PBS (negative control). After incubation at room temperature for 4 h And centrifugation for 5 min at 10,000 g, 100 µl of the supernatant from all samples were transferred to a 96-well plate, and the absorbance was measured using a microplate reader (TECAN Znfinite M200, Austria) at a wavelength of 577 nanometers with 655 nanometers as the reference wavelength. The hemolytic degree was expressed as the hemolytic ratio using the following formula:

$${\rm Hemolysis \:ratio = (OD (test) - OD (negative \:control)) / (OD (positive \:control) - OD (negative \:control)) \times 100\%.}$$

### MTT assay

RPMI-1640 media supplemented with 10% fetal bovine serum (FBS) And 1% penicillin/streptomycin was used to cultivate the CT26 cell line, which was acquired from the Pasteur Institute in Tehran, Iran. CT26 cells were planted in 96-well plates with full culture media in order to measure the IC50 values. For a full day, the cells were exposed to various concentrations (from 5 to 30 µg/mL) of free 5-FU, DA, a 5FU + DA combination, And their nanoliposomal formulations. Following incubation, each well received 100 µL of a newly made 5 mg/mL MTT solution. The medium was carefully removed from the plates after they had been incubated for four hours at 37 °C. After dissolving the formazan crystals in 100 µL of DMSO, they were left to incubate for Another half hour at 37 °C in the dark. To assess cell viability, the optical density (OD) was measured with An ELISA plate reader at 570 And 630 nm.

### Mice and cell lines

Six- to eight-week-old female BALB/c mice were purchased from the Royan Institute in Iran. Mice were acclimated for 7 days pre-experiment to minimize stress-induced variability. Mice were kept in controlled environments with a 12-hour light-dark cycle, constant humidity and temperature, and free access to food and drink. Yasuj University of Medical Sciences’ Institutional Ethics Committee and Research Advisory Committee gave their approval to the experimental procedure (ethics number: IR.YUMS.REC.1401.091).To reduce discomfort, BALB/c mice were sedated prior to tumor implantation. To stimulate tumor growth, 5 × 10⁵ CT26 cells in 60 µl PBS were injected subcutaneously into the right flank after intraperitoneal injection of 12.5 mg/kg xylazine (2%) And 100 mg/kg ketamine (10%). The cells were transported and stored in the cold chain until injection. To maintain genetic stability, the CT26 cell line was used, which had undergone a limited number of passages.

### The development of experimental cancer models

**Fig. 1:**
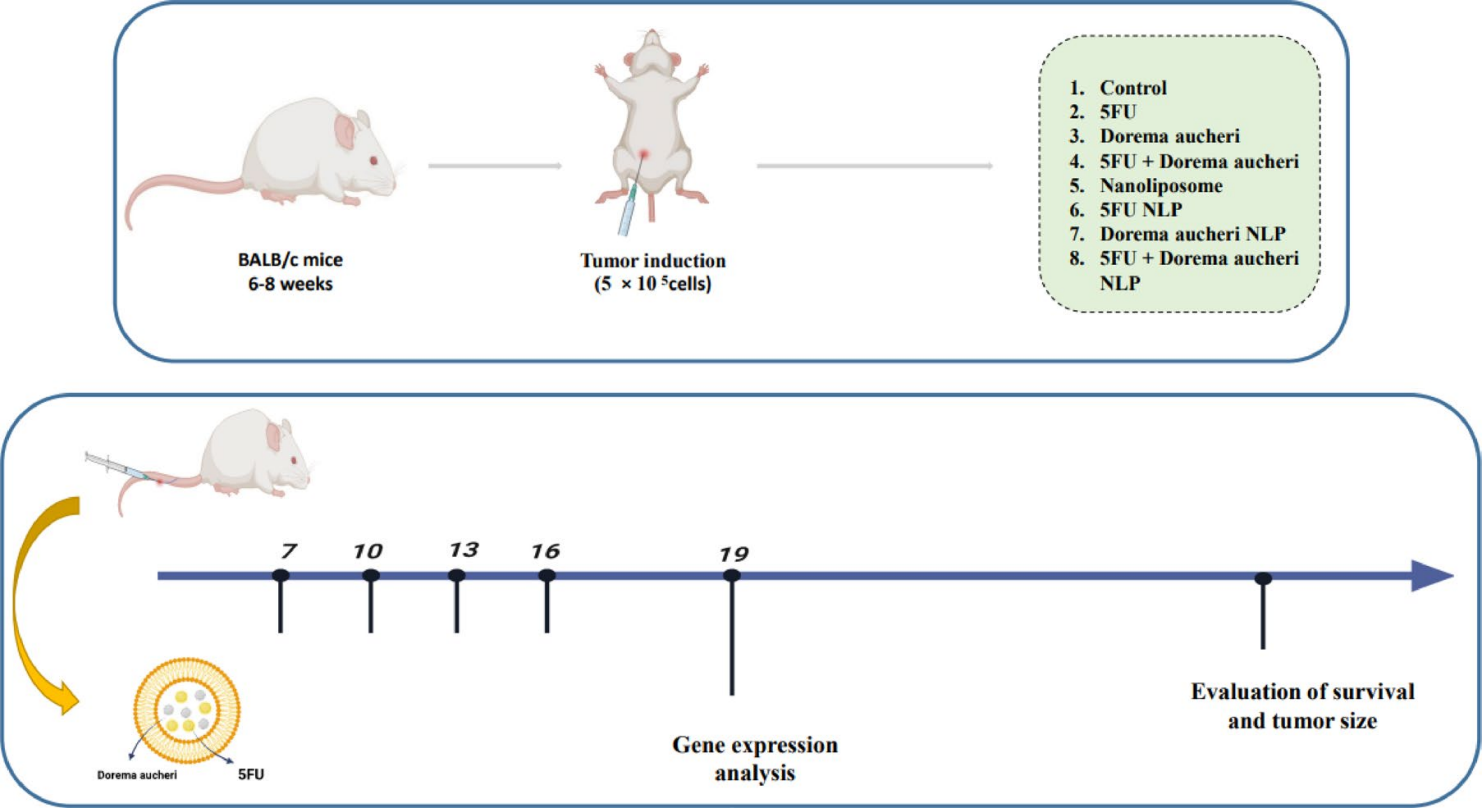
Induction of the model And division of groups And group treatment. 5× 10⁵ CT26 cells were inoculated subcutaneously into the right flank of the mice. On day 7 after inoculation, the mice were randomly divided into 8 groups, with 6 mice in each group. Nanoliposomes containing 5FU And DA were injected intravenously into the mice 4 times as shown in the figure. Finally, the mice were sacrificed three days after the last injection to examine gene expression, and a number of mice were also kept for survival.

Mice with established tumors were randomly assigned to eight treatment groups, each containing 6 mice.According to guidelines and previous studies conducted on this tumor model^35,36^. Based on similar studies^37,38^, a selected dose of 10 mg/kg of each substance was selected. Each group received intravenous injections of 125 microliters) Contains 0.2 mg of each substance **(**of the respective compound, as outlined in Fig. 1. The injections were administered four times, with a three-day interval between doses. Every two days, tumor growth was tracked, and tumor volumes were computed using the formula: tumor volume (mm³) = a² (mm) × b (mm) × 0.52, where ‘a’ denotes the smaller tumor diameter and ‘b’ the bigger one^25^. Three mice per group had their tumors surgically removed on day 19 for additional study. Mice were euthanized by gradual CO₂ inhalation followed by cervical dislocation to ensure death. Similarly, blood samples were collected to examine serum levels of biochemical indicators and their serum was separated.For the survival study, three mice from each group were used^39^. All methods were carried out in accordance with relevant guidelines and regulations, and all methods are reported in accordance with ARRIVE guidelines (https://arriveguidelines.org).

### Real-time PCR

Using real-time polymerase chain reaction (RT-PCR), the expression levels of mRNA for target genes in tumor tissues from different treatment mouse groups were evaluated. Following the manufacturer’s instructions, Trizol reagent was used to isolate total RNA from frozen tissue samples.The purity of extracted RNA was assessed using a Nanodrop device by measuring the absorbance at wavelengths of 260 nm, 280 nm, And 230 nm. The PrimeScript™ RT reagent kit (Takara, Japan) was then used to create complementary DNA (cDNA). Using particular primers, RT-PCR was performed on a Bio-Rad thermocycler using SYBR^®^ Green chemistry in a 20 µL reaction mixture. These primers’ sequences are listed in Table 1. A 15-minute initial denaturation phase at 95 °C was followed by 40 cycles of denaturation (20 s at 95 °C), annealing (30 s at 60 °C), and extension (30 s at 72 °C) in the gRT-PCR methodology. The reference gene used for normalization was beta-actin. A melting curve analysis was carried out to verify the precise amplification of the target genes. The ΔCt technique was used to calculate the relative amounts of mRNA expression.

**Table 1:** Gene expression analysis using primer sequences.

Primers	Sequences
**Beta actin**	Forward: 5′-TGAGCTGCGTTTTACACCCT-3′ Reverse: 5′-TTTGGGGGATGTTTGCTCCA − 3′
**VEGF** (Vascular Endothelial Growth Factor)	Forward: 5′-TTCCTGTAGACACACCCACC-3′ Reverse: 5′-GACATCCTCCTCCCAACACA-3′
**VEGF-R2** (Vascular Endothelial Growth Factor Receptor 2)	Forward: 5′-GTGGTCTTTCGGTGTGTTGC-3′ Reverse: 5′-GTAGGCAGGGAGAGTCCAGA-3′
**VEGF-C** (Vascular Endothelial Growth Factor C)	Forward: 5′-CGCTGTGTCCCATCGTATTG − 3′ Reverse: 5′-AGACTTGGGCCTCTGTTACC-3′
**VE-Cadherin** (Vascular Endothelial Cadherin)	Forward: 5′-CACGGACAAGATCAGCTCCT-3′ Reverse: 5′-CACATAGTGGGGCAGCGATT-3′

#### Statistical analysis

GraphPad Prism version 8 And SPSS version 26 (SPSS, Chicago, IL, USA) were used for statistical analysis. Tukey’s post hoc test was used after a one-way AnOVA to evaluate group differences. Log-rank tests were used to assess Kaplan-Meier survival curves. P-values below 0.05 were regarded as statistically significant.

## Results

### Characterization of NLPs

**Fig. 2:**
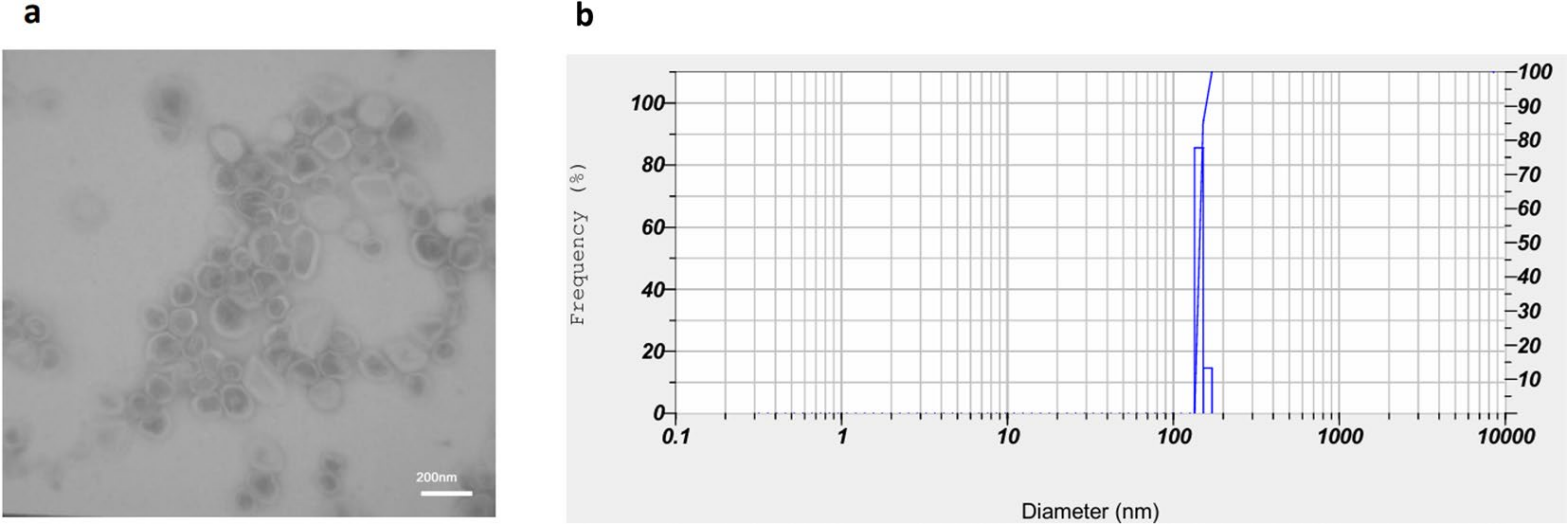
**a**) A TEM image of a nanoliposome containing both 5-FU And DA, with An average size of 200 nm, is presented. **b**) The size distribution of these nanoliposomes, as determined by DLS Analysis, is also shown. The study found uniformly spherical nanoparticles with a hydrodynamic diameter of 146± 4.6 nanometers, a zeta potential of −2.3 millivolts, And a polydispersity index of 0.138. The stability of the nanoparticles was assessed through DLS Analysis And HPLC analysis, showing minimal drug leakage and loading efficiency exceeding 80%.

A TEM microscope was used to examine the morphology and properties of the NLPs, and the results showed that the nanoparticles were uniformly spherical in shape (Fig. 2a) The mean aspect ratio across all the fields was 1.03 ± 0.05, and there was no statistically significant variation between fields (ANOVA, *p* > 0.05), indicative of uniform distribution in shape. The average circularity obtained was 0.87 ± 0.02, further verifying spherical uniformity.

DLS measured the size, zeta potential (ZP), and polydispersity index (PDI) of the nanoparticles. The hydrodynamic diameter was approximately 146 ± 4.6 nanometers, ZP was about − 2.3 millivolts, And PDI was around 0.138 (Fig. 2b). To assess their stability, the NLPs were stored at 4 °C, and their size was monitored over time using DLS analysis. The particle size remained relatively consistent during storage, indicating excellent stability (Table 2). Additionally, minimal drug leakage was observed for both 5-FU and DA from the NLP–5FU–DA formulation (Table 2). HPLC Analysis also showed that the encapsulation efficiency of 5-FU And DA in nanoliposomes was 80± 5.11 And 78± 6.9, respectively.

**Fig. 3:**
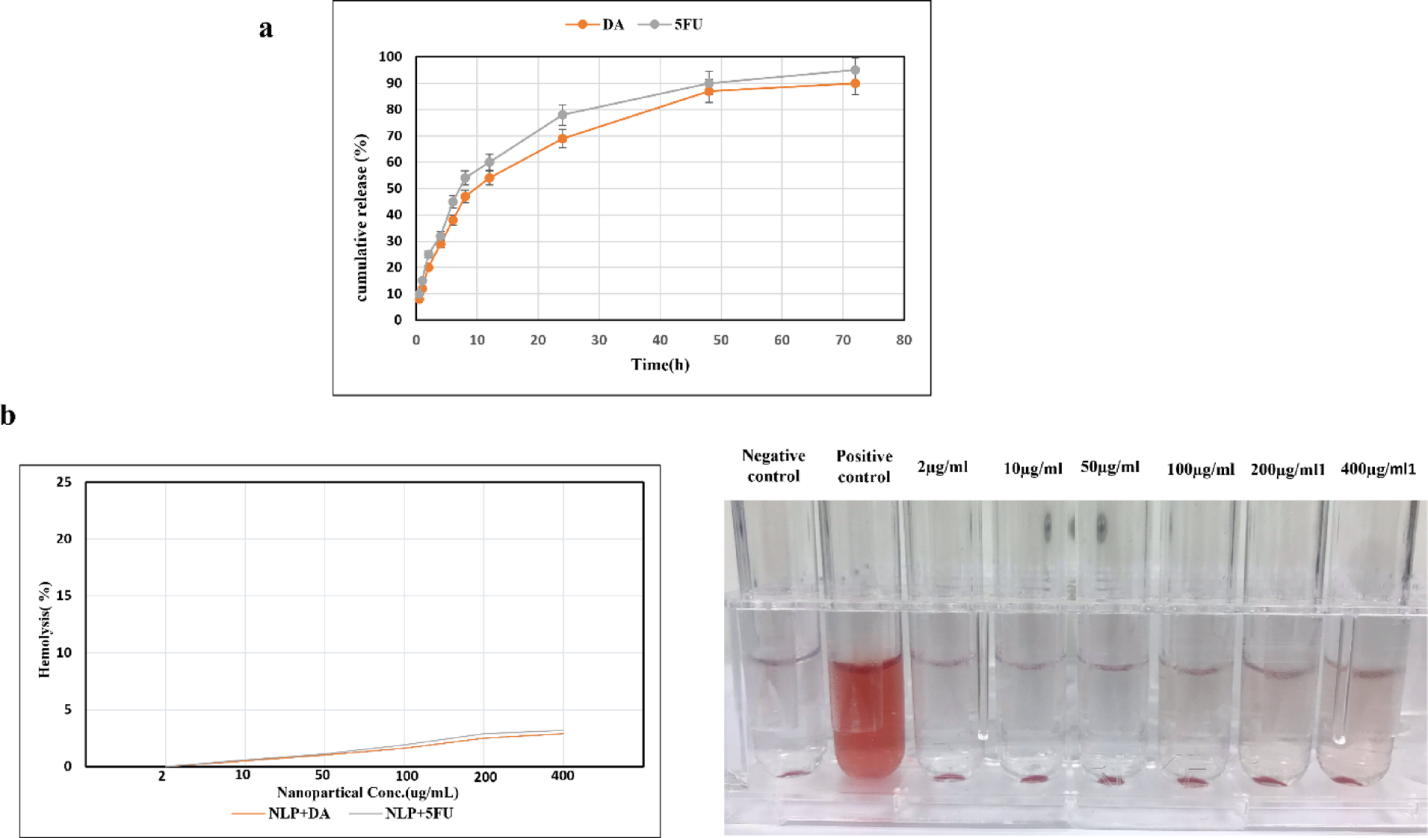
**a**) Release profile of 5FU And DA from nanoliposomes. The release rates of both were almost similar And more than 90% of the drugs were released by 72 h. **b**) Hemolysis assay in three nanoliposome formulations including NLP + DA, NLP + 5FU and NLP + 5FU + DA. As shown, no significant hemolysis was observed for all three compounds at different concentrations.

The drug release profile is shown in the Fig. 3a. As shown, 5-FU exhibited a faster release rate, reaching approximately 95% at 72 h, while DA reached 90% over the same period. However, both 5FU And DA were released in the free state by more than 90% into the medium within the first 6 h. These release profiles prove that encapsulation yields extended drug release, whereas free drugs are released extremely rapidly, a key consideration for the sustenance of therapeutic plasma levels and reduction in systemic toxicity.

We also investigated the hemolysis assay of these nanoparticles. As shown in Fig. 3, it can be concluded from the results that none of the compounds caused significant damage to red blood cells And even at a concentration of 400 µg/ml, the percentage of hemolysis was below 4%.

**Table 2:** This table illustrates how the size And rate of leakage of 5-FU And DA are affected by a three-week storage period at 4 °C.

Storage time (week)	0	1	2	3
Size (nm)	146 ± 4.6	148 ± 4.1	156 ± 6.9	166 ± 8.8
polydispersity index (PDI)	0.138	0.141	0.144	0.147
zeta potential	−2.3	−2.3	−2.1	−2.0
Increase in 5-FU leakage (%)	0	1.8	2.8	3.2
Increase in DA leakage (%)	0	2.4	3.1	4.0

### The MTT assay was used to assess cytotoxicity

**Fig. 4:**
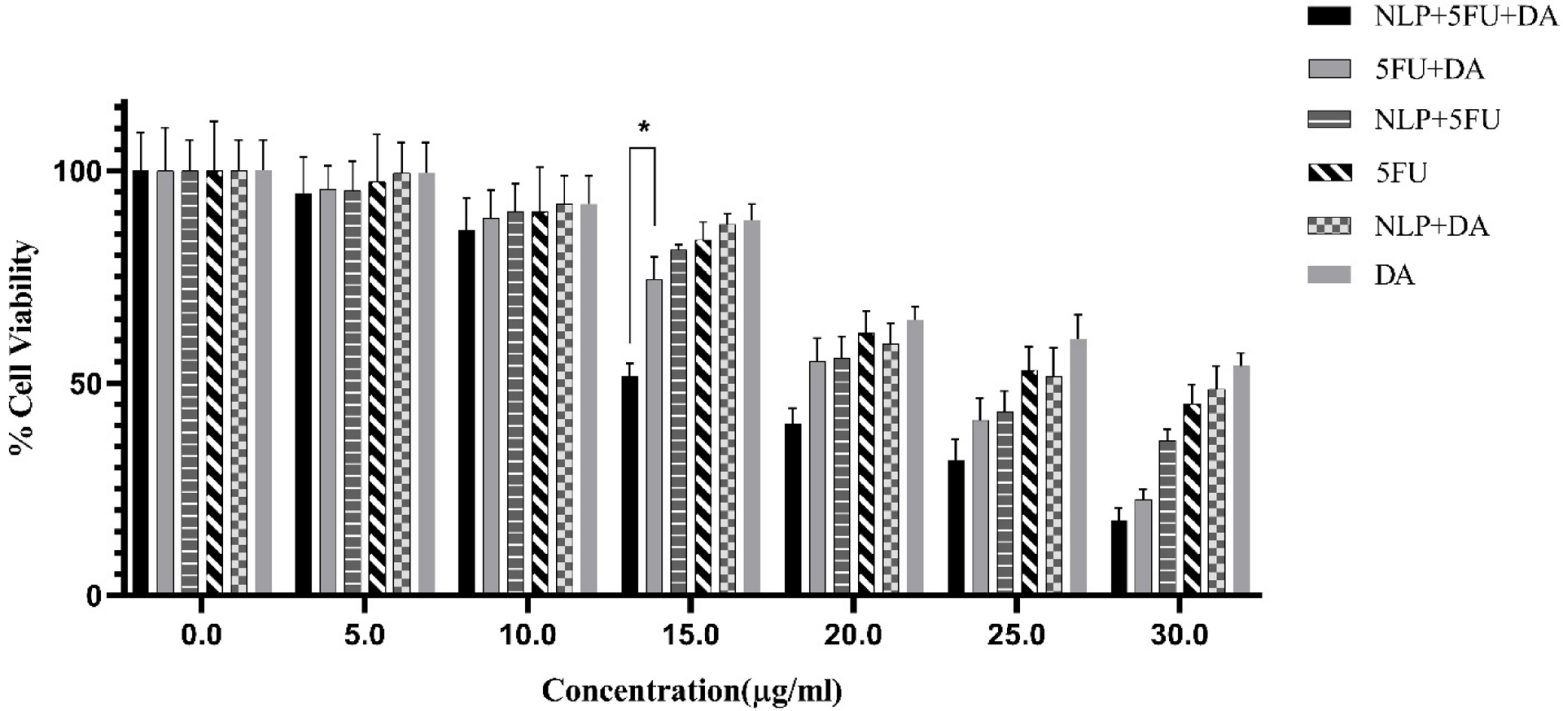
The toxic effect of treatment with different studied groups after 24 h on the CT26 cell Line. Experiments were repeated in triplicate. MTT assay showed that the treatment with either free 5FU + DA or NLP + 5FU + DA inhibited CT26 cell proliferation in a dose-dependent manner. Inhibition of growth in cancer cells was greater when the nanoliposome-encapsulated formulation was compared to the combination of free drugs. The Liposome-encapsulated versions of both 5-FU and DA also inhibited better.

The cytotoxicity of free 5FU + DA versus the form of 5FU + DA encapsulated in nanoliposomes differs significantly, according to the findings displayed in Fig. 4. The MTT assay results indicate that treatment with either free 5FU + DA or the NLP + 5FU + DA reduced CT26 cell proliferation in a dose-dependent manner over a 24-hour period. The IC50 values, which represent the concentration required to inhibit 50% of cell growth, were 21.28 µg/ml for free 5FU + DA And 17.11 µg/ml for NLP + 5FU + DA in the CT26 cell line (*p* = 0.01). These findings suggest that the nanoliposome-encapsulated formulation was significantly more effective at inhibiting cancer cell growth compared to the free drug combination. Although free 5FU + DA was the most potent in reducing cell proliferation compared to other treatment groups (5-FU, DA, 5FU + NLP, DA + NLP), the Liposome-encapsulated versions of both 5-FU and DA also showed superior inhibition of cell growth when compared to their respective free drug forms.

### In tumor-bearing mice, the NLP + 5FU + DA therapy improves tumor regression and boosts survival

**Fig. 5:**
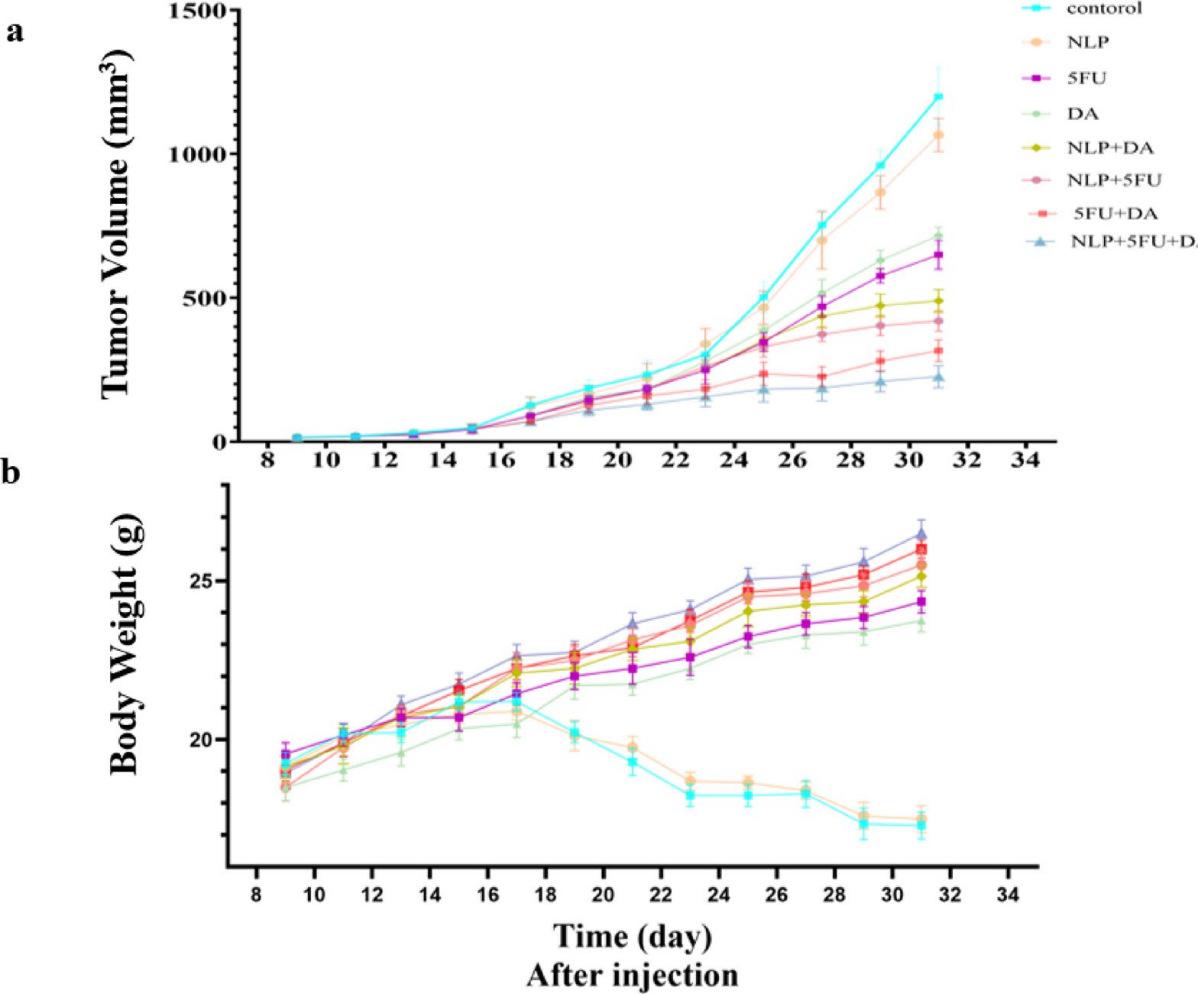
**a**) The tumor growth curve is shown in CT26 tumor-bearing mice after multiple treatments. The study found that tumor volume increased consistently in the control group and treatment groups, with a significantly lower rate in all treated groups. The NLP + 5FU + DA group showed the smallest increase, And the groups receiving the free combination of 5-FU and DA showed the lowest tumor volume growth rates. **b**) Average changes in body weight in mice after multiple treatments (*n* = 6). This study showed that mice with colorectal tumors, especially in the NLP + 5FU + DA group, exhibited An increase in body weight compared to the control group. Similarly, the free combination of 5FU + DA also showed an increase. Similarly, in the control and NLP groups, weight loss was observed as the tumor progressed.

Tumor volume in the control group consistently increased at each measurement time point. A similar trend was observed in the treatment groups, although the rate of increase was significantly lower in all treated groups (*P* > 0.0001). The smallest increase in tumor volume was noted in the NLP + 5FU + DA group. As illustrated in Fig. 5a, the groups that received the free combination of 5-FU and DA, followed by the NLP + DA group and the NLP + 5FU group, all exhibited the lowest rates of tumor volume growth.

In terms of body weight, the average weight of mice with colorectal tumors in the control group decreased over time. In contrast, mice in the treatment groups showed an increase in body weight, with this change being statistically significant compared to the control group (*P* > 0.05). The greatest weight gain was observed in the NLP + 5FU + DA group. As shown in Fig. 5b, the groups treated with the free 5FU + DA combination and the NLP + DA formulation also exhibited more weight gain compared to the other groups.

We also examined the levels of biochemical factors in the serum of mice from different study groups. As shown in Table 3, no significant changes were observed in the levels of these factors and all data were within the normal range according to the instructions of the standard kit used.

**Fig. 6:**
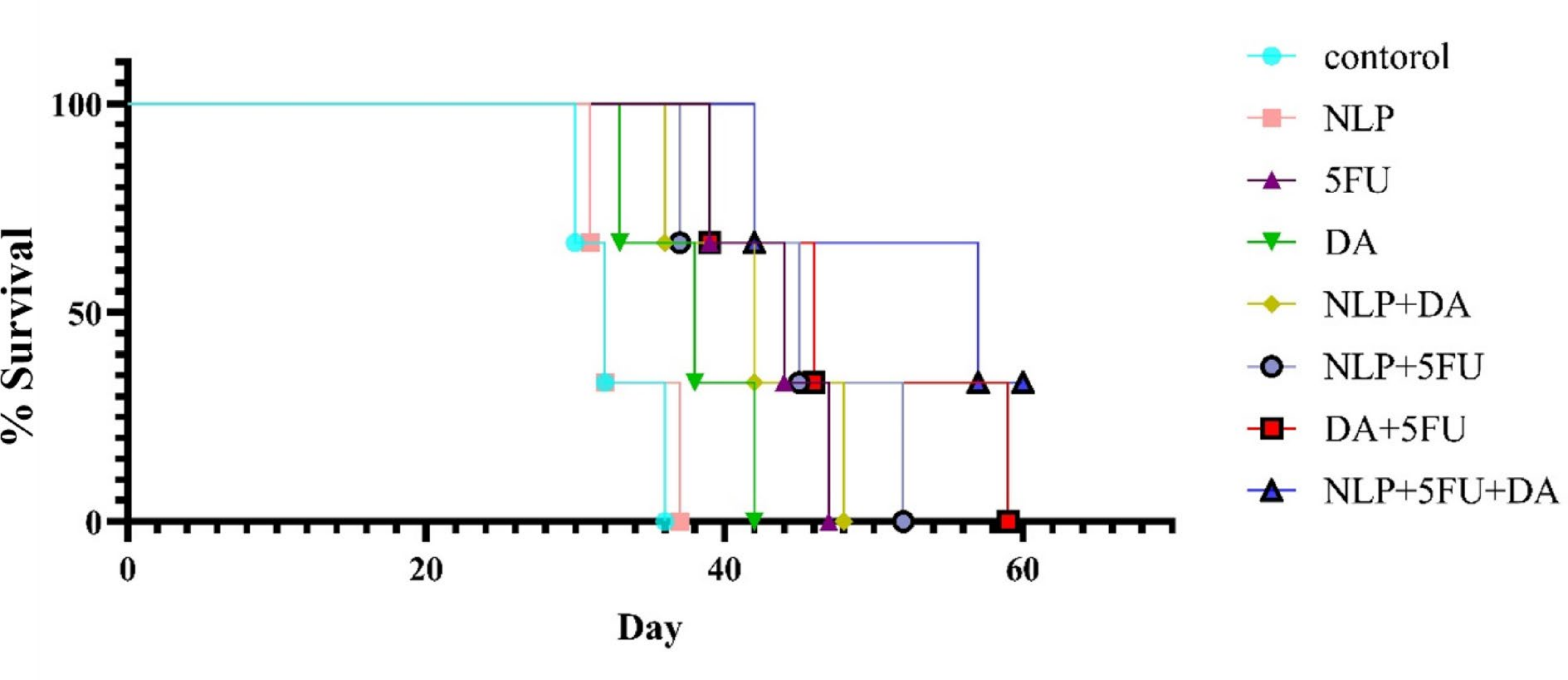
Survival rates of treated mice 60 days following tumor injection. As can be seen in the figure, only in the group NLP + 5FU + DA did one mouse survive after 60 days, And in the other groups, all mice died before day 60. It was significant compared to the control group.

And finally, we measured the survival rate in the remaining mice from each group, as shown in Fig. 6., NLP + 5FU + DA groups had a 33.3% survival rate after 60 days, which was greater than the other groups, And 1 mouse survived (*P* = 0.001).

**Table 3:** Serum levels of biochemical indicators in the different study groups. No significant changes were observed in any of the indicators in any of the groups. (ALT: Alanine aminotransferase, AST: aspartate aminotransferase, ALP: alkaline phosphatase, BUN: blood Urea Nitrogen).

	ALT (U/L)	AST (U/L)	ALP (U/L)	Total Bilirubin (mg/dl)	Albumin (g/dl)	BUN (mg/dl)	Creatinine (mg/dl)
**Contorol**	36 ± 8.3	49 ± 6.8	125 ± 5.7	0.4 ± 0.1	3.3 ± 1.1	11.2 ± 4.9	0.3 ± 0.1
**NLP**	37 ± 7.1	52 ± 7.9	122 ± 8.9	0.5 ± 0.2	3.9 ± 1.4	13.4 ± 3.2	0.4 ± 0.2
**DA**	38 ± 5.9	44 ± 6.4	134 ± 5.8	0.5 ± 0.2	4.1 ± 1.2	12.2 ± 3.1	0.3 ± 0.2
**5FU**	40 ± 6.6	58 ± 8.6	140 ± 4.7	0.6 ± 0.2	3.7 ± 2	11.1 ± 4.5	0.5 ± 0.2
**NLP + DA**	39 ± 7.4	60 ± 5.8	136 ± 6.3	0.4 ± 0.1	3.4 ± 1.8	10.9 ± 5.8	0.4 ± 0.1
**NLP + 5FU**	41 ± 6.2	59 ± 6.9	137 ± 7.9	0.5 ± 0.2	4.2 ± 1.8	11.4 ± 5.2	0.4 ± 0.2
**DA + 5FU**	43 ± 5.1	54 ± 4.9	141 ± 5.6	0.6 ± 0.1	4.9 ± 1.1	15 ± 2.9	0.5 ± 0.1
**NLP + 5FU + DA**	42 ± 4.9	54 ± 6.4	137 ± 7.1	0.5 ± 0.1	4.5 ± 1.2	14.8 ± 3.4	0.5 ± 0.2

### Gene expression

**Fig. 7:**
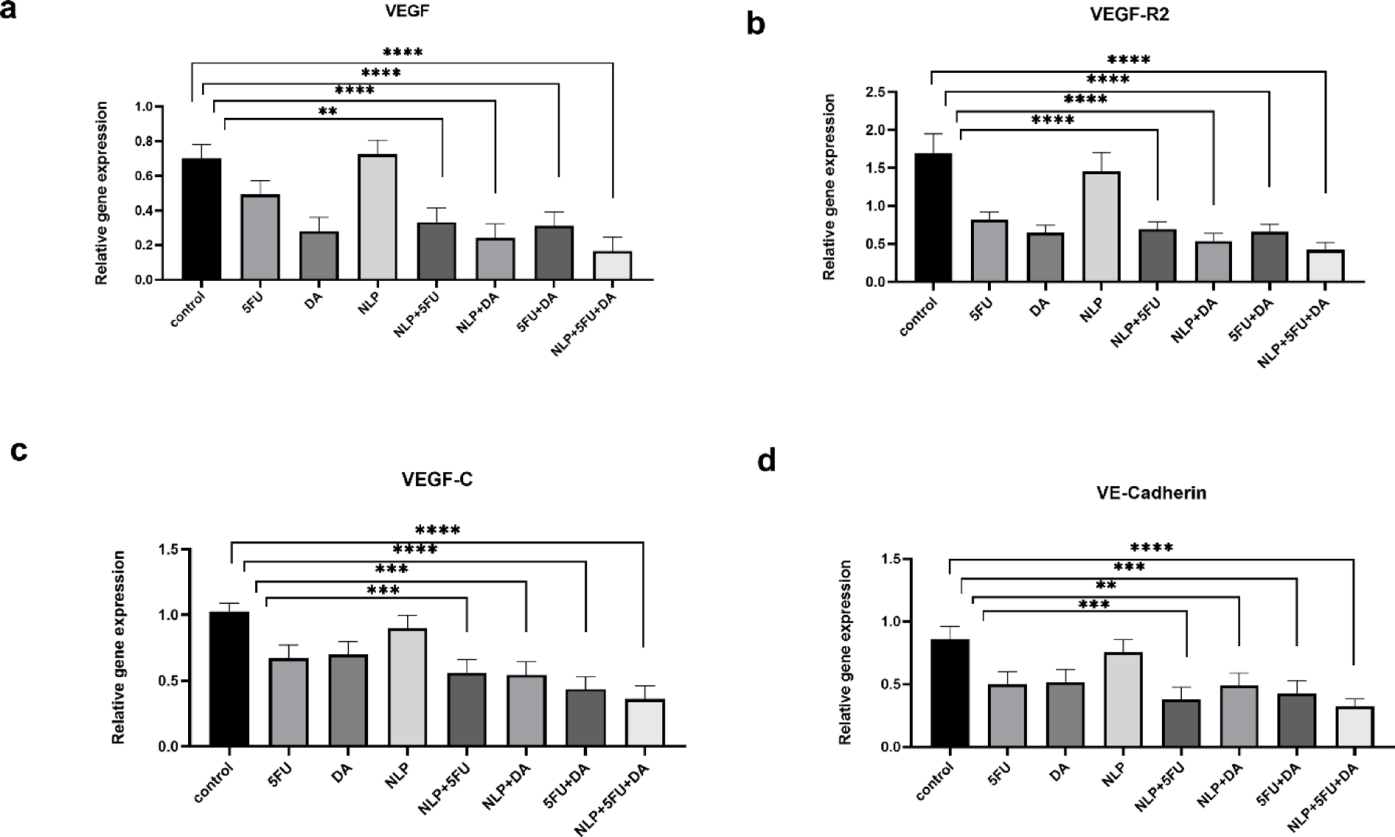
This figure presents the Analysis of the real-time PCR data. It was found in the research that the groups that received the nanoliposomes with 5-FU and DA had significantly lower levels of gene expression compared to the control group and the other treatment groups. The reduction was higher with the liposomal combination. The reduction was also noted with the single drug preparations. Data are presented as means ± SDs; * *P* < 0.05, * * *P* < 0.01, and * ** * *P* < 0.0001.

As shown in Fig. 7, the expression levels of all four genes were significantly lower in the groups treated with nanoliposomes containing 5-FU And DA compared to both the control group And other treatment groups. Similarly, the group receiving the free combination of 5-FU and DA also showed a significant reduction in gene expression, although the decrease was more pronounced in the liposomal combination. In contrast, the groups treated with each compound alone did not exhibit significant changes in gene expression. However, the liposomal formulations of these individual drugs did result in a notable reduction compared to the control group, although the decrease was less than that observed with the combined liposomal treatment.

## Discussion

Herbal medicines are often used to minimize side effects and enhance the effectiveness of traditional cancer treatments. These plant-derived treatments are not a substitute for conventional cancer treatments but are being investigated for their ability to complement other cancer treatments^40,41^. Given that DA has antioxidant activity, and antioxidants may help reduce oxidative stress and potentially alleviate some chemotherapy side effects, as well as anticancer effects in previous studies^24,25^, it may improve the efficacy of 5-FU through potential synergistic effects. Our research revealed that the Bilhar extract displays substantial anti-tumor properties in mice with CT26 cell line tumors. The extract’s cytotoxicity increased in a dose-dependent manner when loaded onto nanoliposomes. In this in vivo study, the extract caused a reduction in tumor size in mice, with a more pronounced effect observed when it was encapsulated in nanoliposomes. This extract has been shown to exhibit similar anti-cancer effects against other cancer cell lines, including MCF7, OCC-O2, HepG2, and A549. Therefore, the present study is the first study on the CT26 cell line in comparison with studies conducted in this field^14,42–44^. Active components in medicinal plants, including Bilhar, can induce apoptosis in cancer cells and thereby have antitumor activity^45,46^. On the other hand, nanotechnology, particularly nanoliposomes, has become crucial in improving the delivery and enhancing the effectiveness of herbal medicines in cancer therapy. Nanoliposomes are lipid bilayer vesicles with nanosize that can contain herbal medicines and improve their bioavailability, solubility, and stability and thus solve the problem of poor bioavailability and stability of these compounds. This approach allows for targeted delivery of drugs to tumor tissues, reducing the impact on healthy cells and minimizing side effects^47–49^. In the present study, the liposome form of DA had more cytotoxicity than its free form, and it also caused a further reduction in tumor growth in the mouse model. These results were similar to our previous study on the OCC-02 cell line^44^. Given that DA has antioxidant activity, and antioxidants may help reduce oxidative stress and potentially alleviate some chemotherapy side effects, as well as anticancer effects in previous studies^24,25^, it may improve the efficacy of 5-FU through potential synergistic effects.

5-FU belongs to a group of chemotherapy drugs known as antimetabolites^50^. Nanoliposomes, given the characteristics mentioned above, can be used to enhance the therapeutic effects of 5-FU. Encapsulating 5-FU in nanoliposomes aims to increase its efficacy while minimizing adverse effects on healthy cells^51^. Researchers have found that adding 5-FU to a nanoliposomal formulation can make the drug more effective against tumors and improve treatment outcomes^52^. The results of our study showed that the Liposome form of 5-FU has more cytotoxicity than its free form and also causes a greater reduction in tumor volume in mice. In a similar study, Liposomal nanoparticles loaded with 5-FU were synthesized. This formulation showed more cytotoxicity in vitro than the free form of the drug. In studies using a mouse model with PDX, animals treated with the Liposome form had a tumor volume that was more than 9 times smaller than those treated with the free form^53^.

In the present study, we loaded 5-FU and DA in a liposome; our results showed that this formulation significantly inhibits the growth of CT26 cancer cells in vitro, and its cytotoxicity is significantly higher than the free form of these compounds. Similarly, in vivo, it caused a significant decrease in tumor growth. In general, our results indicate that the combination of chemotherapy and herbal medicine (5-FU and DA) increases the antitumor effects. Similar to our results, other studies also showed the results have reported the same in combined treatment. The study conducted by Luput et al. on the Liposomal combination of 5-FU and simvastatin demonstrated that combined treatments based on liposomal formulations enhance antitumor activities when compared to combined treatments with free drugs. This treatment showed antitumor activity in mice with colorectal cancer, mainly through inhibition of tumor angiogenesis^54^. Dual drug-loaded liposomes (FC-DP-Lip) encasing CUR And 5-FU were made using the thin-layer hydration approach in the study by Liu et al. The results demonstrated that dual nanoliposomes can enhance the toxicity of 5-FU to cancer cells, and these nanoliposomes were cytotoxic against a variety of cancer cell types^55^. The studies mentioned are similar to our study in that they include 5-FU, but the combination drugs are different. These studies show that combining other methods with 5-FU can provide more acceptable results.

Finally, in this study, we investigated the effect of these interventions on the expression of the VEGF, VEGF-R2, VE-Cadherin, and VEGF-C genes in the tumor microenvironment. These genes play an essential role in the pathogenesis of CRC by promoting angiogenesis, regulating signaling pathways, and contributing to the aggressive nature of some colorectal tumors. Their interactions and dysregulation in CRC highlight their importance as potential targets for therapeutic interventions and prognostic markers in the management of this disease^56,57^. Our results showed that 5-FU and DA, both in free form and liposome, significantly decrease the expression of these genes. However, the greatest decrease in expression was seen when the treatment was used in combination, especially in liposome form (NLP + 5FU + DA). In previous studies, it has been shown that treatment with 5-FU leads to a significant decrease in angiogenesis, VEGFR2, and VEGFR1 gene expression, which indicates that 5-FU can affect the expression of VEGF-related genes And pathways. Furthermore, 5-FU has been shown to inhibit cell proliferation and directly downregulate VEGF-A expression, highlighting its role in modulating VEGF levels. Our goal was to investigate the expression of these genes throughout the combined medication treatment, which differed from past research in this area.

The specific effect of 5-FU on the expression levels of VE-Cadherin And VEGF-C is not clearly discussed in the provided references. However, since these genes are involved in Angiogenesis, the modulation of angiogenesis by 5-FU can indirectly affect their expression And function. Finally, it can be said that 5-FU is due to its ability to reduce angiogenesis, modulating pathways related to VEGF, and its effect on key genes involved in cancer progression can play a prominent role in the treatment of CRC^58–61^.

This study’s limitations include using only one cell line and cancer model. It is suggested that different cell lines be examined and further studies be conducted on the tumor microenvironment and the impact of this combined treatment on the immune cell profile within the tumor microenvironment. Ultimately, if satisfactory results are obtained after these stages, the study can be continued as a small clinical trial.

## Conclusion

Our findings showed that a combination of chemotherapy and herbal drugs (5FU + DA) can be well loaded into liposome nanoparticles and these nanoliposomes have suitable physicochemical and biodegradable properties. The synthesized nanoparticles had beneficial antitumor effects in reducing tumor growth and survival of a mouse model of colorectal cancer compared to monotherapy or free form of these compounds. The combination also showed beneficial effects in reducing the expression of angiogenesis-related genes at the tumor site.

## Data Availability

This article contains all of the data created or examined during this investigation. Further inquiries can be directed to the corresponding author.

## Abbreviations

CRC: colorectal cancer
5-FU: 5-Fluorouracil
DA: Dorema aucheri
NLP: Nanoliposomes
CT26: Colon Tumor 26
VEGF: Vascular Endothelial Growth Factor
VEGF-R2: Vascular Endothelial Growth Factor Receptor 2
VEGF-C: Vascular Endothelial Growth Factor C
VE-Cadherin: Vascular Endothelial Cadherin
HPLC: high-performance liquid chromatography
DLS: dynamic light scattering
TEM: transmission electron microscopy
